# Fluorine‐contained hydroxyapatite suppresses bone resorption through inhibiting osteoclasts differentiation and function in vitro and in vivo

**DOI:** 10.1111/cpr.12613

**Published:** 2019-04-10

**Authors:** Shibo Liu, Hao Zhou, Hanghang Liu, Huanzhong Ji, Wei Fei, En Luo

**Affiliations:** ^1^ State Key Laboratory of Oral Diseases National Clinical Research Center for Oral Diseases, Department of oral and Maxillofacial Surgery, West China Hospital of Stomatology, Sichuan University Chengdu China; ^2^ Department of Stomotology Sichuan Academy of Medical Science & Sichuan Provincial People's Hospital Chengdu China

**Keywords:** bone resorption, fluorohydroxyapatite, osteoclast, osteoporosis

## Abstract

**Objectives:**

Fluorine, an organic trace element, has been shown to unfavourably effect osteoclasts function at a low dose. Use of hydroxyapatite (HA) has been effective in exploring its roles in promoting bone repair. In this study, we used HA modified with fluorine to investigate whether it could influence osteoclastic activity in vitro and ovariectomy‐induced osteoclasts hyperfunction in vivo.

**Materials and methods:**

Fluorohydroxyapatite (FHA) was obtained and characterized by scanning electron microscope (SEM). Osteoclasts proliferation and apoptosis treated with FHA were assessed by MTT and TUNEL assay. SEM, F‐actin, TRAP activity and bone resorption experiment were performed to determine the influence of FHA on osteoclasts differentiation and function. Moreover, HA and FHA were implanted into ovariectomized osteoporotic and sham surgery rats. Histology and Micro‐CT were examined for further verification.

**Results:**

Fluorine released from FHA slowly and sustainably. FHA hampered osteoclasts proliferation, promoted osteoclasts apoptosis, suppressed osteoclasts differentiation and function. Experiments in vivo validated that FHA participation brought about an inhibitory effect on osteoclasts hyperfunction and less bone absorption.

**Conclusion:**

The results indicated that FHA served as an efficient regulator to attenuate osteoclasts formation and function and was proposed as a candidature for bone tissue engineering applications.

## 1 INTRODUCTION

The dynamic process of bone remodelling is maintained through tight intercoordination between bone formation and bone absorption owing to the activities of osteoblasts and osteoclasts.^1^, ^2^ The exquisite balance can be interrupted result from osteoclasts hyperfunction triggered by various pathologic conditions, including inflammation, metabolic and endocrine disorders.^3^, ^4^ Patients with osteoporosis, diabetes and Paget's disease suffer from accelerated bone destruction and pathological bone loss.^5^, ^6^, ^7^ Osteoclasts are multinucleated giant cells exactly verified to degrade bone solely and essential for maintaining mineral homeostasis.^8^, ^9^ Increased number or function of osteoclasts cause bone absorption to prevail over formation, leading to not only bone microstructural deterioration and increased bone frailty, but also biomaterial implants failure.^10^, ^11^, ^12^

Hydroxyapatite (HA), an inorganic component in bone tissues, provides most of bone mechanical properties (eg strength and stiffness). With micro‐sized pores and grains as well as high biocompatibility, HA has been efficaciously used as bone graft substitute or fillings for biomedical applications.^13^, ^14^ However, HA is vulnerable under poor osteogenesis condition in osteopenic animals, and thus, the osteoporotic state throws down the gauntlet to the wide application of HA.^15^ Osteoclasts with increased function are capable of resorbing mineralized structures, causing stability reduction and subsequent loosening of implant.^16^ Therefore, osteoclasts could be as a cellular target to attenuate the imbalance and avoid excessive bone loss under osteoporotic conditions.^17^

Fluorine, an indispensable trace element belonging to halogens group, was necessary for human health, such as nervous regulation and dental caries prevention. Moreover, fluorine exerts positive actions on maintaining bone structure and function.^18^ Fluorine bounds to a mitogenic stimulus for osteoblasts and possesses the capability to promote osteoblasts proliferation, enhance bone formation.^19^ Furthermore, studies have shed light on the preventive effects of fluorine on osteoclastogenesis through restraining osteoclasts formation and decreasing expressions of matrix metalloproteinase 9 and tartrate‐resistant acid phosphatase.^20^ Titanium surface modified with fluoride could increase the synthesis of osteoprotegerin, and impeded osteoclasts activation and differentiation were followed.^21^

In this study, fluorohydroxyapatite (FHA) was obtained through modifying HA with fluorine. Our aim was to examine whether fluorine‐modified HA could still suppress osteoclasts function in vitro. Its effects on regulating bone metabolism in osteoporotic rat model in vivo were also explored, expecting to provide a therapeutic agent for more stable implants under osteoclasts hyperfunction conditions.

## 2 MATERIALS AND METHODS

### 2.1 Preparation and characterization of FHA

Fluorohydroxyapatite was obtained from Engineering Research Center for Biomedical Materials of Ministry of Education, East China University of Science and Technology. Prior to detections and applications, the discs experienced ultrasonic cleaning of distilled water and autoclaving. Scanning electron microscope (SEM, KYKY‐2800, Beijing, China) was utilized for FHA morphological examination. Before observation, the samples were dehydrated and gold‐plated. Energy dispersive spectrum (EDS; Oxford Instruments, Abingdon, Oxfordshire, UK) was used to analyse the contents of constituent elements in the micro‐area of materials. X‐ray diffraction (XRD) patterns of FHA (FHA‐1 and FHA‐2) and HA were obtained by powder XRD instrument (Bruker, Karlsruhe, Germany).

### 2.2 Release experiment

To appraise the release behaviour fluorine from FHA, five samples were respectively placed in a polyethylene plastic bottle containing 5 mL deionized water and tightly closed, new soaking water was replaced per day during immersion process. Fluorine selective electrode (Mettler Toledo Zurich, Switzerland) was used to measure fluorine concentration for 21 days. Following measured three times, the average values were taken and the sustained‐release curve of fluorine was plotted (per day release and corresponding cumulative release), and the values were corrected via comparing to HA in the same situation.

### 2.3 Cell culture and reagents

Mouse macrophage cell line RAW 264.7 was obtained from ATCC (No: TIB‐71, Manassas, VA, USA). Cells were cultured in α‐MEM medium supplemented with 10% foetal bovine serum (FBS; Gibco BRL, Grand Island, NY, USA), 50 U/mL penicillin and 50 μg/mL streptomycin (Gibco BRL, Grand Island, NY, USA) at a humidified incubator (37℃, 5%CO_2_). RANKL was prepared in advance from Peprotech, Rocky Hill, NJ, USA.

### 2.4 Osteoclasts differentiation, proliferation and apoptosis

To test the effect of FHA on osteoclasts differentiation, FHAs discs were placed in 6‐well plate, and RAW264.7 cells were seeded at the concentration of 3 × 10^3^/well. Waiting for one hour to assure the cells settle on FHAs, certain amount of medium was added. Twenty‐four hours later, cells were treated with 50 ng/mL of RANKL for 6 days. Equally, HA served as control. After osteoclasts were induced, SEM observation was carried out. Concisely, FHAs and HAs were taken out and rinsed three times in phosphate‐buffered saline (PBS), then the discs were soaked in 2.5% glutaraldehyde sustaining 15 minutes for fixation. Afterwards, the discs were dehydrated through an ethanol series of different concentrations (50%, 75%, 80%, 95% and 100%) and air‐dried. Definitively, samples were observed under SEM after sputter coated with gold and visualized.

To test the effect of FHA on cell proliferation, MTT assay was performed. Briefly, osteoclasts were obtained and seeded in 24‐well plate with 2 × 10^5^/well, and FHAs were preconditioned with medium containing 10% FBS and then placed. Cells co‐cultured with HA without fluorine served as control. After incubated for 1, 2, 3, 4, 5, 6 and 7 days, cells were harvested and subjected to MTT assay (Sigma‐Aldrich, St. Louis, MO, USA) according to the manufacturer's protocol. Beckman DU7400 spectrophotometer (Beckman coulter, Miami, FL, USA) was used to measure the MTT absorption.

TUNEL assay was performed to identify apoptotic cells using One Step TUNEL Apoptosis Assay Kit (Beyotime, Shanghai, China). Osteoclasts were induced as described previously. Thereupon, TUNEL staining was operated in accordance with manufacturer's instructions at 24 and 48 hours in the presence of HA and FHA, respectively, after osteoclasts formed. Fluorescence microscope (Leica, Wetzlar, Germany) was applied to detect the cells, and the percentage of TUNEL‐positive cells was calculated.

### 2.5 Bone resorption experiment

Bone resorption experiment was taken to evaluate the negative function of FHA on osteoclasts function. RAW264.7 cells were induced into osteoclasts via RANKL as described previously. Mature osteoclasts were collected and cultured in 24‐well plates at 2 × 10^3^/well in α‐MEM medium supplemented with 10% FBS in the presence of HA and FHA for about six days. Cells were obtained from the materials and implanted on bovine femur wafers (6 × 6 × 0.2 mm^3^). Changing medium every 3 days, the bone wafers were collected after 10 days, dried and sputter coated with gold for SEM detect. Randomly selecting 10 views from each wafer to examine the quantity and area of bone lacunae.

### 2.6 TRAP activity analysis and F‐actin immunofluorescence staining

To assess the formation of osteoclasts, tartrate‐resistant acid phosphatase (TRAP) activity was measured via a leucocyte acid phosphatase kit (Sigma‐Aldrich). Cells were incubated in the presence of FHA and HA in medium environment containing RANKL for 6 days. Before staining, cells were washed from the materials and cultured in culture plates. Cells were then washed with PBS for three times and fixed utilizing 4% paraformaldehyde for 15 minutes. Washing with PBS three times again was followed. The experiment was performed according to the protocol. The number of osteoclasts per disc was quantified and averaged. F‐actin staining was then performed to investigate FHA effect on F‐actin ring formation as previously reported. Mature osteoclasts were seeded on glass coverslips. Cells were then fixed with 4% formaldehyde in PBS for ten minutes and permeabilized using ice‐cold acetone. Coverslips were subsequently incubated with rhodamine phalloidin stock solution (Cytoskeleton, Denver, CO, USA) for 20 minutes and observed using a confocal imaging system.

### 2.7 In vivo animal studies

#### 2.7.1 Ovariectomized rats model

Thirty‐two six‐month‐old male healthy Wistar rats weighing 200‐250 g were obtained from West China Medical Laboratory Animal Center, Sichuan University. All animal procedures were approved by the Animal Care Committee of Sichuan University. Rats with intact jaw and normal occlusion were given one‐week adaptive phase. For ovariectomy (n = 16), prior to surgery, the rats were anesthetized with 10% chloral hydrate through intraperitoneal injection at 3 mL/kg body weight. Skin was prepared about 2 × 2 cm^2^size. Two‐centimeter‐long incision was operated in the midline of the back, subcutaneous tissue was separated, and rat ovary was found at the intersection of the psoas and the costal margin and spayed after being ligating the oviduct. The contralateral ovary was removed via the same method, and muscles and skin were layered and sutured. For sham surgery (n = 16), all processes were  the same as ovariectomy group but no ovarian resection. Postoperatively, penicillin was intramuscular injected at 200 000 IU/time, 2 times/d for 3 days posterior to surgery.

#### 2.7.2 Mandibular defects

Thirty days after surgery, mandibular defects were established in all rats. Briefly, anaesthesia and preoperative preparation were performed. Parallel to the lower edge of the mandible, 1.5‐cm‐long incision was established. Skin, subcutaneous tissue and periosteum were completely removed to completely expose the mandible angle region. Then, a 5‐mm‐diameter circular bone defect was accomplished with drills and accepted HA insertion in left jaw defects. FHA was inserted in the contralateral defect via the same way. Crosscut was layered suture. Postoperative intramuscular injection of penicillin was implemented at 200 000 IU/time, twice a day for three consecutive days.

#### 2.7.3 Histological processing

Bilateral mandibles were collected from rats after one and 4 weeks. The 1‐week samples were subjected to TRAP staining, and the 4‐week samples were for haematoxylin and eosin (H&E) staining and Masson's trichrome staining. Briefly, all samples were fixed with 4% paraformaldehyde solution, decalcified with 10% EDTA at 37°C for three weeks. Afterwards, samples were dehydrated with alcohol gradient and embedded in paraffin, and 5‐μm‐thick sections were sliced. The sections were prepared with haematoxylin and eosin (H&E) staining and Masson staining for further histological examination.

#### 2.7.4 Micro‐CT scanning analysis

Four‐week bone samples were stored for Micro‐CT study. Briefly, the samples were scanned by Micro‐CT (μ‐CT80; SCANCO, Zurich, Switzerland), operating voltage and current were 70 kV and 114 μA, respectively, and integration time was 700 ms. To select the area centring on material as volume of interest and observe new bone regeneration with 2048 × 2048 pixels, trabecular thickness (Tb.Th, μm) and trabecular number (Tb.N, 1/mm) were calculated.

### 2.8 Statistical analysis

Quantitative data are presented as mean ± standard deviation, and comparisons between groups were analysed by two‐way analyses of variance (ANOVA). The experimental results were statistically analysed using SPSS 13.0 statistical software. *P* values were considered statistically significant when less than 0.05.

## 3 RESULTS

### 3.1 Characterization of FHA

**Figure 1:**
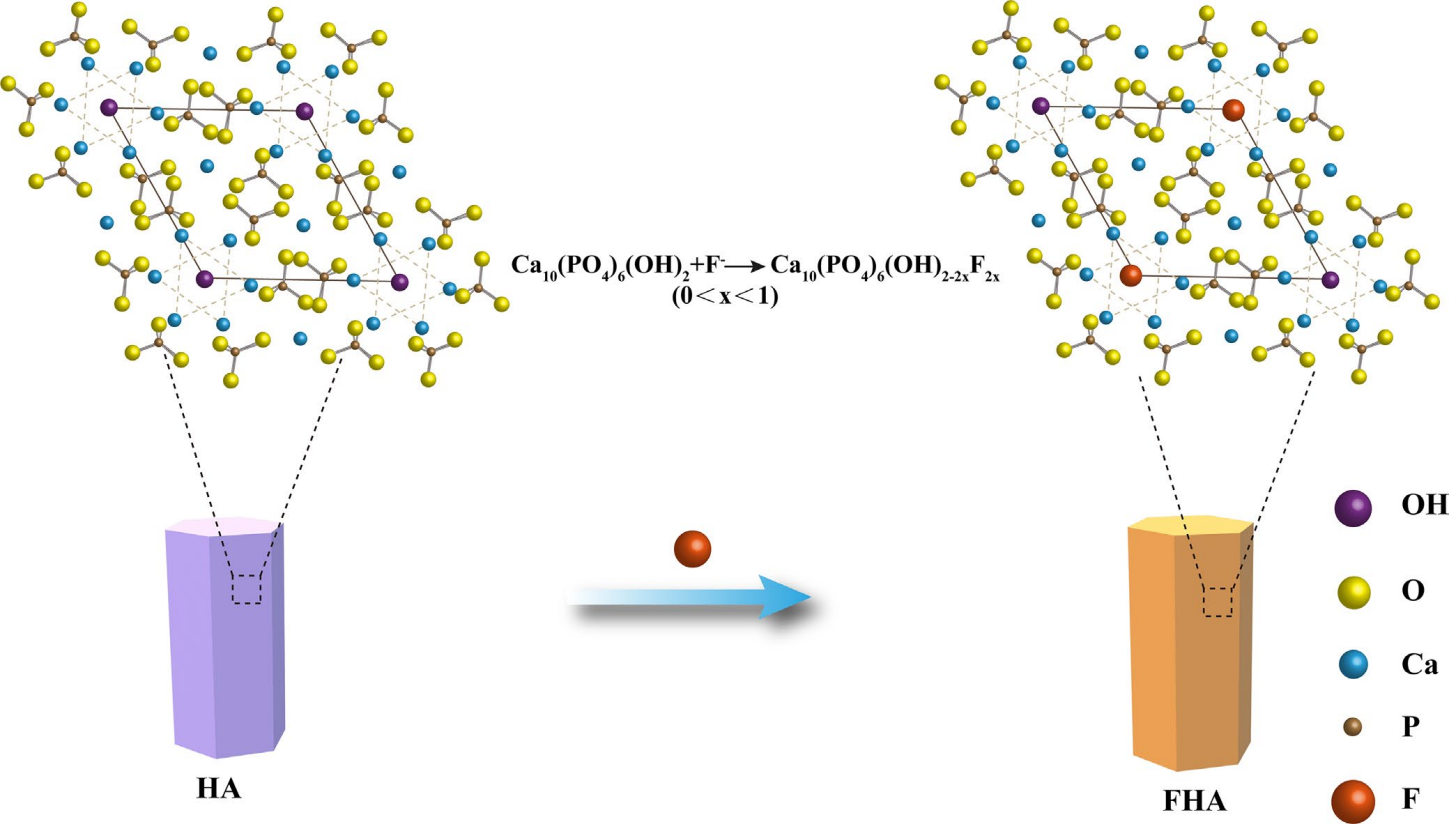
The sketch of fluorohydroxyapatite (FHA) structures (plane projection): Fluorine partially replaced the hydroxyl in hydroxyapatite (HA)

**Figure 2:**
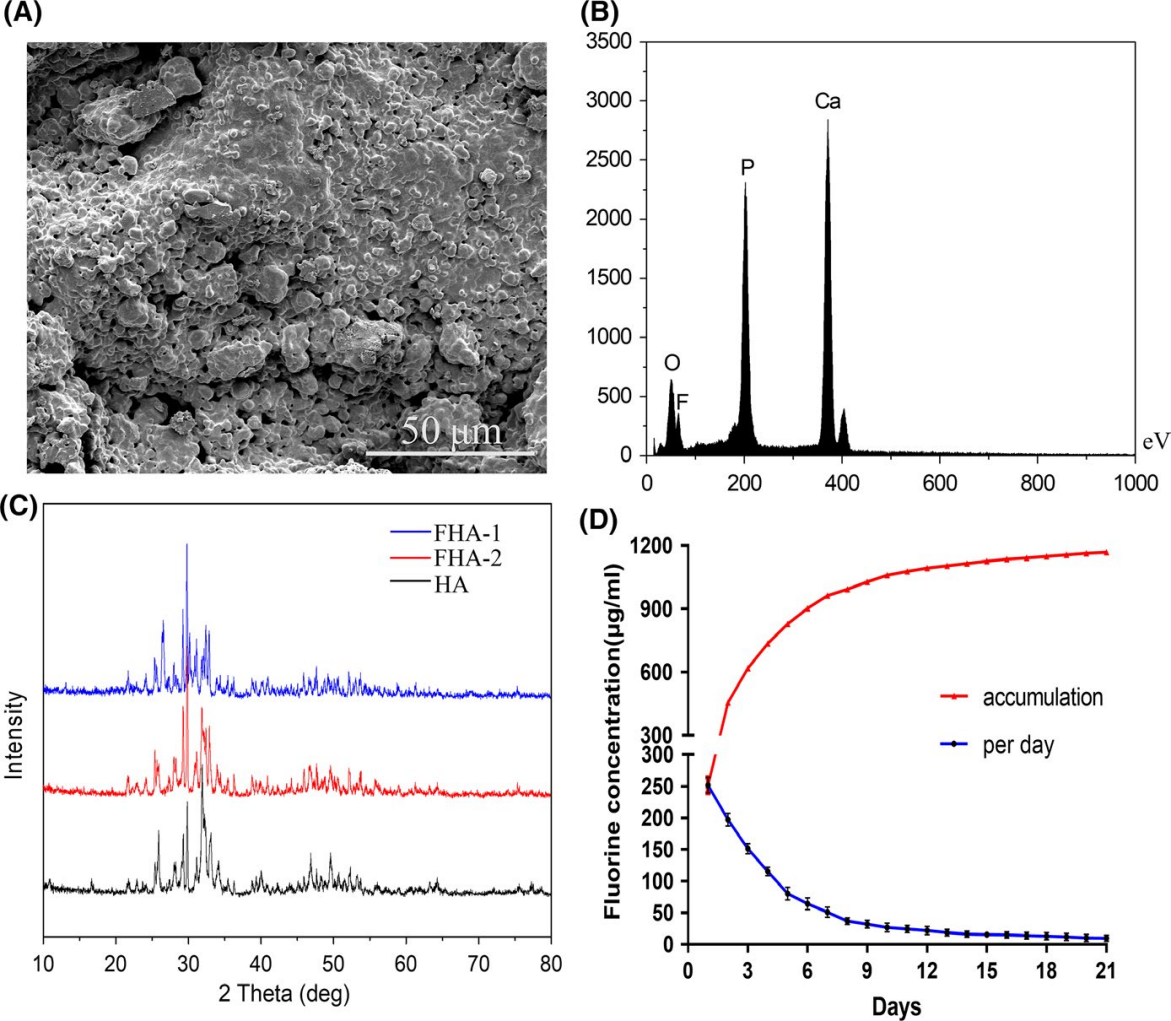
Characterization of FHA. A, presented the SEM image of FHA with porous morphology. B, EDS analysis illustrated the presence of fluorine element in FHA, including fluorine element. C, XRD patterns of FHA and HA. D, Fluorine released from FHA at a relatively slow speed and maintained a certain concentration per day, the gross concentration increased with the constant fluorine release

Fluorohydroxyapatite was prepared and its structure was shown in Figure 1. The synthesized FHA depicted a tough morphology and has a porous microstructure (Figure 2A). The EDS analysis of FHA identified the presence of Ca, O, P and F (Figure 2B), confirming that fluorine has successfully combined with HA. Additionally, XRD detect results indicated that FHA patterns were in accordance with pure HA (Figure 2C).

### 3.2 Fluorine released from FHA sustainably

The fluorine concentration was detected every day, and release curve was gained according to the measured values (Figure 2D). Per day fluorine concentration got lower with the prolongation of time. During the first ten days, fluorine released relatively fast and had a higher concentration. Then, release speed became slower, per day fluorine release still existed, and the concentration entered a stable period which kept small near 10 μg/L. The values in the text were finally corrected compared to control group. Apparently, the gross amount of fluorine accumulated over the time increased gradually.

### 3.3 FHA containing fluorine inhibited osteoclasts proliferation and differentiation

**Figure 3:**
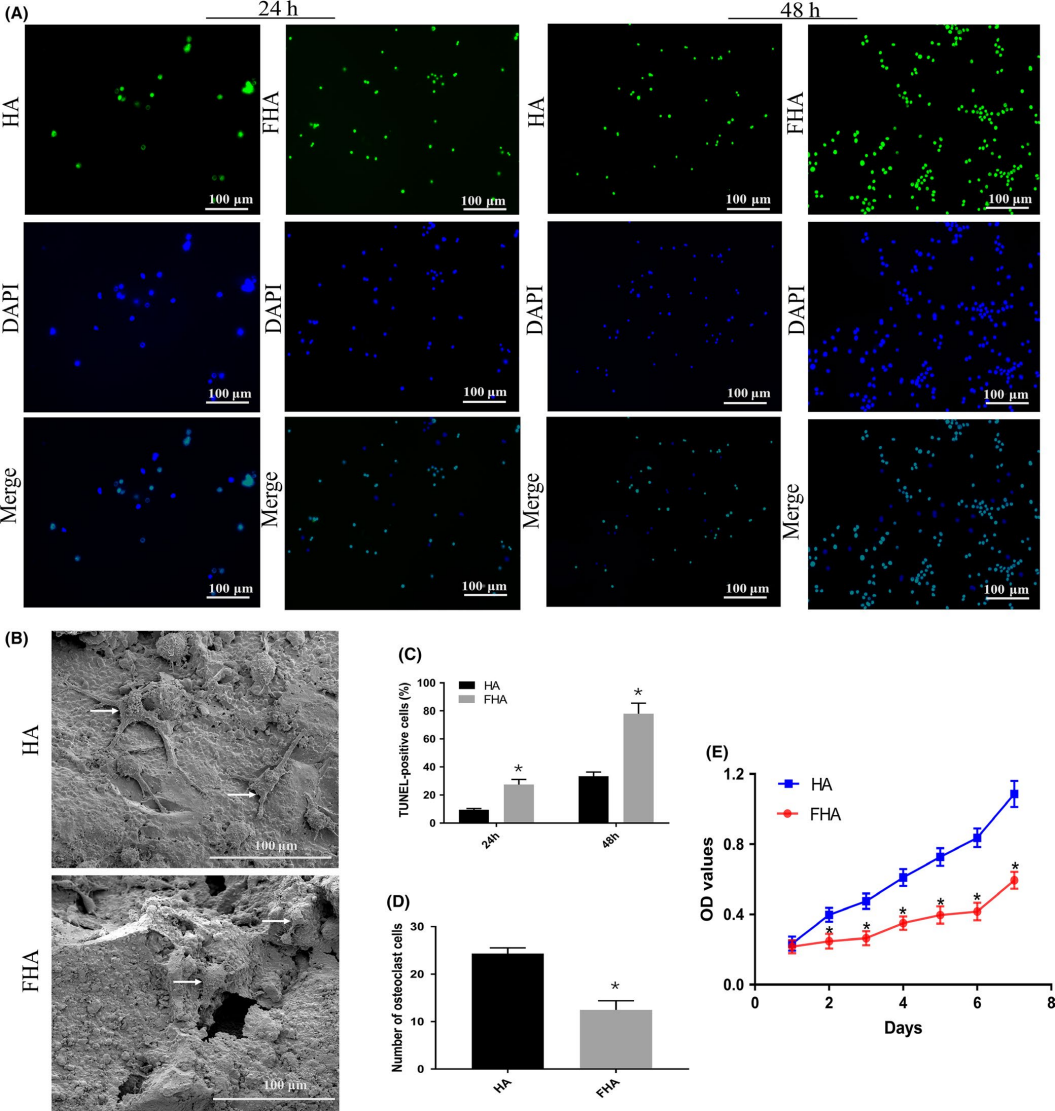
Fluorohydroxyapatite inhibited osteoclasts proliferation and promoted apoptosis. A, Representative images of TUNEL assay at 24 and 48 h, respectively, after osteoclasts were induced. B, SEM images of osteoclasts on HA and FHA, the white arrows indicated the osteoclasts. C, TUNEL staining results shown significantly more apoptotic cells in FHA group. D, Significantly less number osteoclasts were formed in FHA group. E, Induced osteoclasts were treated with fluorine‐contained FHA and fluorine‐free HA, respectively, and were then exposed to MTT assay. *: differences were considered to be statistically significant (*P* < 0.05)

TUNEL assay was subsequently performed to investigate the effect of FHA on osteoclasts apoptosis. As shown in Figure 3A, cells treated with FHA were more positive to TUNEL reaction at 24 and 48 hours, respectively. Cells co‐cultured with pure HA showed a less number in DNA fragmentation, which was obvious via a significant decrease in TUNEL‐positive cells suggesting cell death by apoptosis (Figure 3C), indicating that FHA had a positive impact on osteoclasts apoptosis.

RAW264.7 cells were cultured with RANKL for 6 days without and with materials and subjected to SEM. From the SEM images view (Figure 3B), osteoclasts were large‐size, irregular‐shape and there were pseudo‐feet contacting with the material surface. Morphology difference between FHA and control group was little, yet osteoclasts on FHA were fewer compared with control (Figure 3D), indicating that the presence of FHA inhibited osteoclasts differentiation.

Effects of FHA on osteoclasts proliferation were measured by MTT assay for consecutive 7 days. Proliferation curve was displayed as Figure 3E. Cell proliferation was obviously negatively regulated by FHA compared to HA.

### 3.4 FHA inhibited osteoclasts function

**Figure 4:**
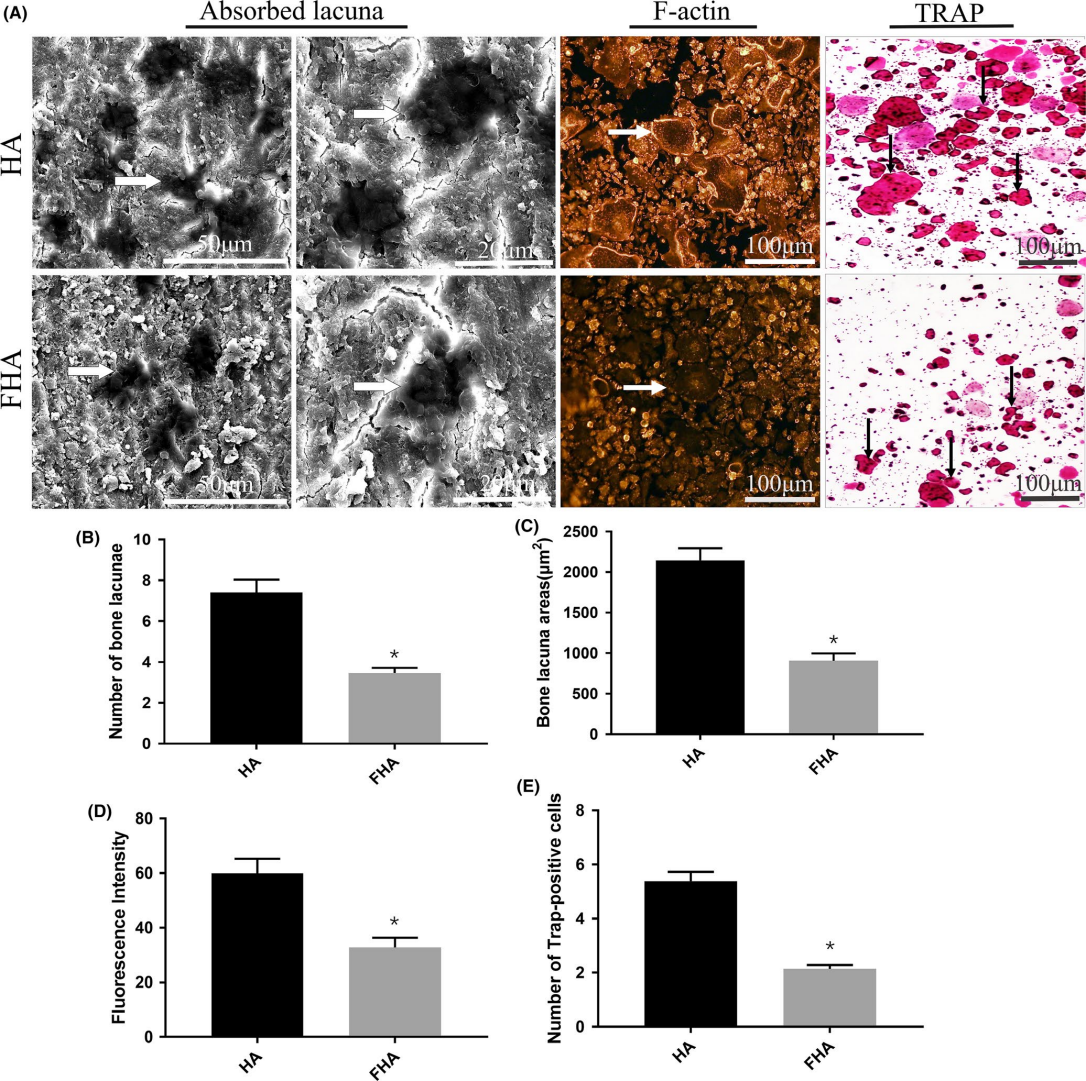
FHA inhibited osteoclasts formation and function. A, Representative images of bone resorption experiment (white arrows, bone lacunae), F‐actin immunofluorescence staining (white arrows, F‐actin ring) and TRAP staining (black arrows, TRAP‐positive osteoclasts); B, Number of bone lacunae in FHA group was significantly lower than HA group; C, Bone lacunae area in FHA group was significantly lower than HA group; D, Fluorescence intensity of F‐actin staining was compared between FHA and HA group. Significant difference was observed. E.TRAP‐positive cells in FHA group had a statistical difference from HA group. *: differences were considered to be statistically significant (*P* < 0.05)

Bone resorption experiment was adopted after osteoclasts were induced. Less number and area of absorbed bone lacunae were observed on FHA group than control group. The difference was statistically significant. F‐actin and TRAP were specific marker for mature osteoclasts. After treated with FHA, the size and morphology of the F‐actin ring were reduced. Fluorescence intensity in FHA was significantly lower than HA group. Equally, the number of TRAP‐positive multinucleated cells was significantly less than that of the composite HA group (Figure 4A‐E). It is inferred that HA combined with fluorine could still regulate osteoclasts function negatively.

### 3.5 FHA could be as an effective participant to inhibit ovariectomy‐induced osteoclasts hyperfunction in vivo

**Figure 5:**
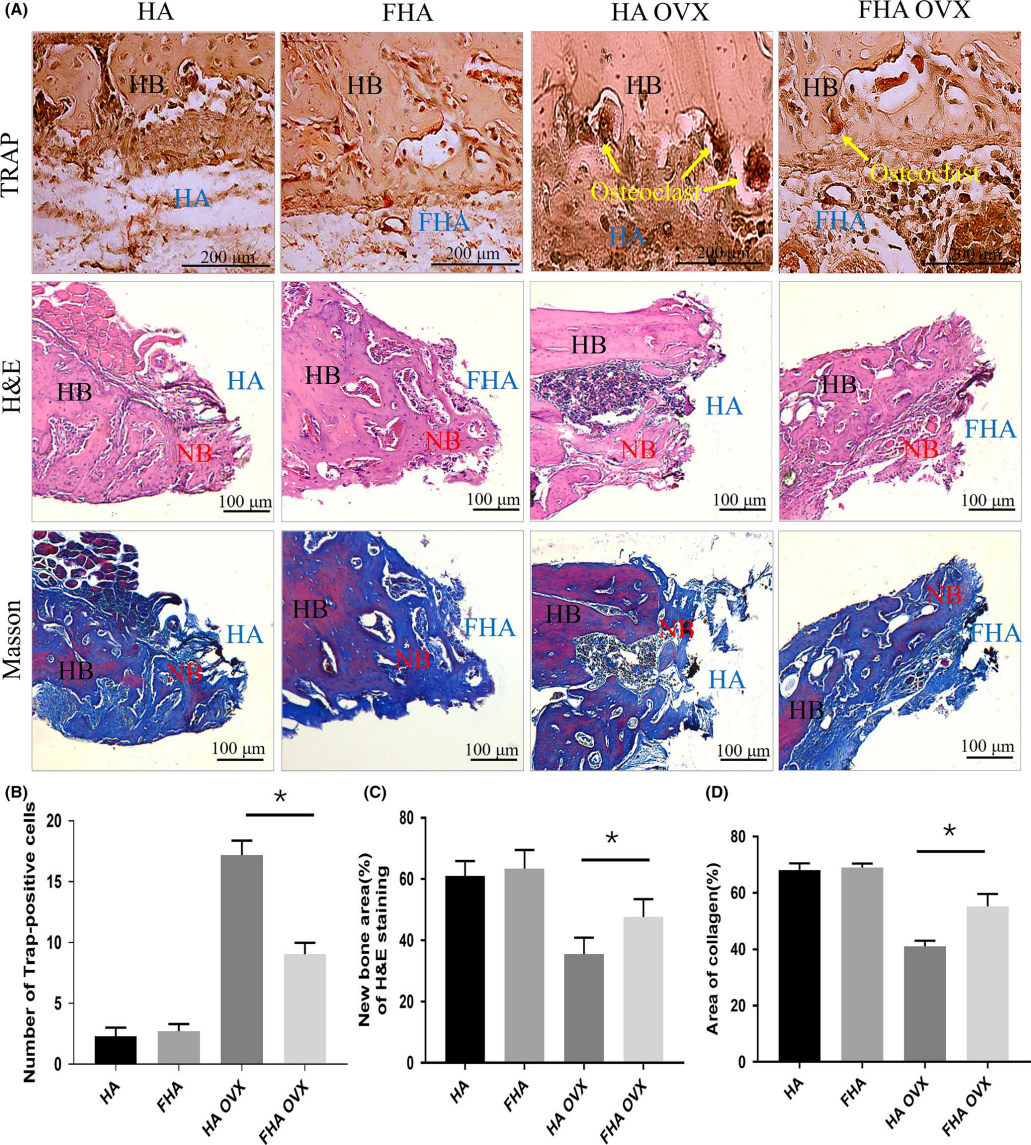
Fluorohydroxyapatite inhibited osteoclasts formation and function in vivo. A, Representative images of TRAP, H&E and Masson staining. (HB, host bone; NB, new bone) B, TRAP‐positive cells were significantly different between HA OVX and FHA OVX groups. C, New bone formation of H&E staining in HA OVX group was less than FHA OVX significantly. D, Collagen formed of Masson staining in FHA OVX group was significantly more than HA OVX group. *: differences were considered to be statistically significant (*P* < 0.05)

Tissue surrounding the mandibular defects was obtained after one week and subjected to TRAP staining. FHA inhibited osteoclasts formation, as evidenced that stronger staining for TRAP activity in the HA OVX group was discovered than in the FHA OVX group. TRAP‐positive osteoclasts cells were not easily found in the sham surgery groups (HA and FHA groups). Tissues around materials were tested by H&E and Masson staining after four weeks. Obvious amounts of new bone around the materials in the sham surgery groups were showed, where there was no statistical significance. Meanwhile, FHA OVX group had significantly more new bone and collagen formation, which exhibited more new bone tissue in contrast to the HA OVX group, demonstrating that FHA had an obvious effect on preventing osteoclast‐mediated bone resorption (Figure 5A‐D).

**Figure 6:**
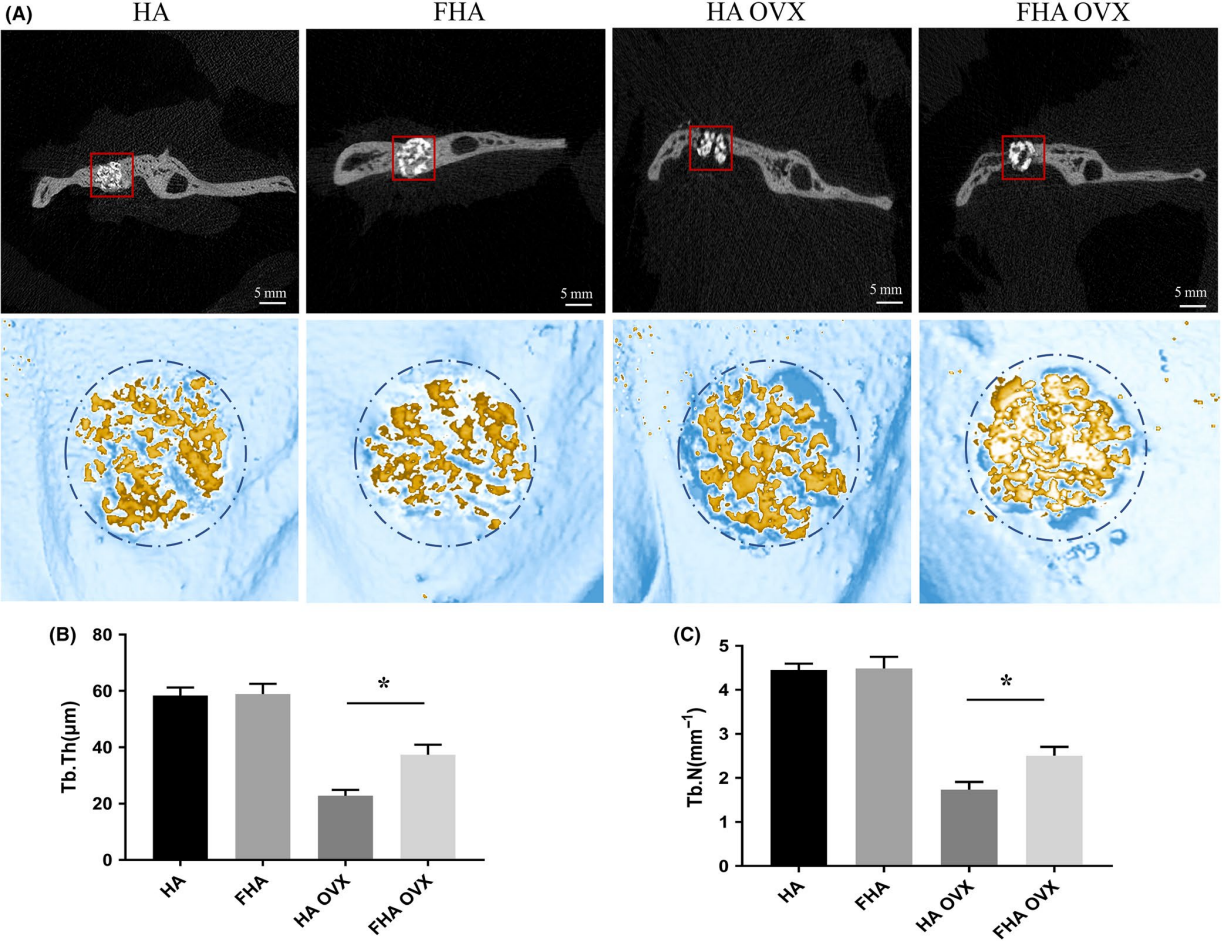
Fluorohydroxyapatite inhibited excessive bone resorption induced by osteoclasts hyperfunction. A, Bone tissue in the mandible bone defect was observed by Micro‐CT and three‐dimensional (3D) images were acquired and utilized for quantitative evaluation by the manufacturer's morphometric software (VGStudio MAX). Red boxes referred the interested areas. B,C, Trabecular thickness (Tb.Th, μm) and trabecular number (Tb.N, 1/mm) of HA, FHA, HA OVX and FHA OVX groups were calculated. *: differences were considered to be statistically significant (*P* < 0.05)

Micro‐CT revealed a significant higher bone volume in FHA OVX group when compared with HA OVX group. Sham surgery rats showed massive bone formation, the materials and tissue were mutual mixed. For the OVX groups, gap was observed between material edge and bone tissue as predicted, yet less was found in FHA OVX. The results were confirmed via software analysis. The 3D‐structure analysis revealed that FHA could retard bone resorption triggered by ovary‐removal‐induced osteoclasts hyperfunction. The function was visually clear in various bone morphometric parameters such as trabecular thickness (Tb.Th) and trabecular number (Tb.N) (Figure 6A‐C).

## 4 DISCUSSION

Hydroxyapatite is vulnerable when exposed to hyperfunction of osteoclasts.^22^ In this study, fluorine‐modifying HA was utilized to determine its regulating effects on osteoclasts function in vitro and osteoporosis‐induced osteoclastic hyperactivity in vivo. A convincing evidence was provided that FHA had an inhibitory effect on osteoclastic activity and suppressed bone resorption in OVX rats via comparison to pure HA. The differences of bone mass formed between FHA and HA group might be due to reduction and diminished function of bone resorption cells (osteoclasts) via FHA suppression.

Bone disorders such as osteoporosis have become one of health challenges to the quality of life.^23^ They are always characterized by increased osteoclasts formation.^24^ Osteoclasts hyperfunction could then increase the risk rating of poor bone healing and fractures.^25^, ^26^ Moreover, generalized osteoporosis was reported to negatively influence implant stability and implant failures are mostly due to poor bone quality.^27^, ^28^ In recent years, scaffolds combined with specific actives substance (eg vascular endothelial growth factor and bone morphogenetic protein 2) were utilized to regulate cell behaviour in acellular therapy to promote bone formation.^29^, ^30^, ^31^ Nevertheless, native proteins which are applied are prone to degrade at a relatively rapid speed in humoral environment.^32^, ^33^ Furthermore, HA has been explored for various biomedical applications and extensively used in bone tissue engineering. As a temporary matrix, HA is able to render a micro‐environment for cell proliferation, differentiation and extra‐cellular matrix deposition owing to its porous microstructure.^34^, ^35^ However, the unstability of HA under osteoporosis states brings it a considerable scope for improvement.^15^

Fluorine, as an active and trace element, widely distributes in nature and is essential to human and animals.^36^ Researches have gradually demonstrated that fluorine is inevitable for bone health. In the human body, approximately 99% of fluorine is associated with the skeletal tissues.^37^ The hydroxyl ion in the HA of bone could be replaced with fluorine when the element incorporates into the apatite lattice during bone formation, and the apatite solubility could be decreased in the presence of fluorine. As a native element, fluorine could avoid the possibility that certain active proteins are deactivated or degraded too quickly.

Fluorine was reported to act on the Jun N‐terminal kinase (JNK) and p38 MAPK signalling pathways which influenced cell proliferation, differentiation and apoptosis.^38^ Relationship between fluorine and bone constituent cells was explored. Fluoride‐modified titanium increased Runx2 expression of osteoblast‐like MG63 cells.^39^ Alkaline phosphatase activity (ALP), a marker for bone mineralization, was enhanced when osteoblasts were exposed to the fluoride‐containing bioactive glasses, suggesting fluorine promotes osteoblast differentiation.^40^ Fluorine promoted the phosphorylation of Akt at serine 473 and GSK‐3β at serine 9, GSK‐3β activity was inhibited and Wnt/β‐catenin signalling was activated, and osteoblast phenotypic genes (Runx2, ALP, osteonectin) were then increased.^41^ BMP/Smads signalling was also proved to be associated with fluorine‐induced osteoblast differentiation.^42^ Moreover, drinking water which was fluoridated could notably depress the hydrocortisone‐induced bone resorption.^43^ Pei Junrui et al found that osteoclasts function in resorption decreased remarkably when fluorine was at a low dose(0.5 mg/L), and the effects became further significant when fluorine concentration increased in a suitable range, pointing out that fluorine concentration was negatively correlated with osteoclasts effect.^44^ Furthermore, there are correlations with fluorine and expression of some proteins/factors involved in osteoclast‐relatively functions.^45^, ^46^ Lower TRAP mRNA levels were detected during healing process after fluoride‐modified titanium implants were inserted into rabbits. Osteoclast‐associated factors and matrix metalloproteinase9 (MMP9) mRNA expression were diminished during osteoclasts differentiation maturity. Also, fluorine could also decrease the osteoclastic bone resorption by inhibiting the expression of nuclear factor of active T cells c1(NFATc1).^47^ TGFβ/TβR1/Smad3 pathway was deemed to be related to the moderating effect on osteoclast by fluorine.^48^ Fluorine inhibited the Sema4D/Plexin‐B1/RhoA/ROCK signalling pathway and enhanced promoted TGF‐β1 expression, which results in up‐regulated osteoprotegerin and inhibited osteoclast differentiation due to the competition between osteoprotegerin and RANK.^49^

In this study, FHA was obtained via modifying HA with fluorine. Scaffold is capable of providing sufficient structural integrity and surface area for interaction between cell and materials.^50^ The characterization of FHA revealed that it showed a porous structure to offer a three‐dimensional architecture for cell spreading. Fluorine could release sustainly from FHA at a relatively low dose. MTT and TUNEL assay demonstrated that FHA had a detrimental effect on cell proliferation. Through bone resorption experiment, F‐actin and TRAP staining, we concluded that FHA material reduced the differentiation and function of osteoclasts cells.

Basing on the above in vitro studies, the role played by FHA for bone formation in vivo was studied. OVX animals are extensively used for osteoporosis model that are characterized by poor bone formation owing to osteoclastogenesis. In the present study, ovariectomized rats were used. We found that the new bone formation in FHA was significantly higher than HA group in OVX rats, suggesting that inhibited osteoclastogenesis was relevant. On the other hand, an important finding was that FHA exerted no evident effect in normal situation (sham surgery groups).

Since it was found that FHA suppressed bone resorption through inhibiting osteoclasts differentiation and function. The underlying other mechanisms remain to be determined. The osteoblasts increased activity in the process was left a matter for future consideration. Whether FHA in inflammation or other metabolic disease state could play the same role is unknown.

In conclusion, FHA has a positive effect on suppressing bone resorption, providing a better understanding of FHA function in acting on osteoclasts function. For the clinical application, the results address the potential and the feasibility of therapeutic interventions involving the fluorine‐containing HA. The material may help in the future to reduce bone resorption, ensure a balanced bone turnover and to generally improve implant stability or skeletal health.

## CONFLICT OF INTEREST

No competing interests exist.
